# Functional nutritional rice: current progresses and future prospects

**DOI:** 10.3389/fpls.2024.1488210

**Published:** 2024-11-19

**Authors:** Sumei Duan, Hao Ai, Shengqin Liu, Aifeng Zhou, Yuhong Cao, Xianzhong Huang

**Affiliations:** ^1^ Center for Crop Biotechnology, College of Agriculture, Anhui Science and Technology University, Chuzhou, China; ^2^ Anhui Xin Fu Xiang Tian Ecological Agriculture Co. Ltd., Ma’anshan, China; ^3^ Ma’anshan Agriculture and Rural Bureau, Ma’anshan, China

**Keywords:** functional nutritional rice, mineral biofortification, breeding, functional food, resistant starch, micronutrients

## Abstract

More than half of the world’s population relies on rice as their staple food for three meals a day. From a dietary perspective, rice can be considered the most important grain in the world. With the continuous improvement of people’s living standards, the demand for food has gradually shifted from being full and eating well to being nutritious and healthy. Developing functional nutritional rice has become an important research direction and strategic initiative for developing a major food concept. In this paper, we review the current progress in the breeding of functional nutritional rice and mineral-biofortified rice. This review focuses on the following aspects: (i) the concept, rice basic structure, nutritional components, and categorization of functional nutritional rice; (ii) genes that have been applied and identified so far, including nutritional functional rice genes, mineral bioenhancement-related genes, and their regulatory mechanisms; (iii) based on the history and technical mainline of rice breeding, research progress in nutritional functional rice using conventional breeding, a combination of conventional breeding and marker-assisted breeding, mutagenesis breeding, genetic engineering technology, and gene editing technology. Based on the current research and industrialization issues, we highlight an outlook of the problems and future developmental directions in nutritional functional rice research.

## 1 Introduction

Since the 1970s, people’s eating habits, dietary structures, and lifestyles have increasingly improved with the development of science and technology and the social economy, as well as the improvement of living standards. However, numerous challenges, such as nutritional imbalance, which has led to a sharp increase in a sub-healthy population, have emerged. Research on functional nutritional rice has attracted the attention of researchers and gradually developed, and has become a key research subject in rice research (Graham et al., 2001). In 2002, the large-scale international cooperation project for the global development of functional rice initiated by the Consultative Group on International Agricultural Research (CGIAR) was implemented. The China National Rice Research Institute joined this international cooperation project in 2003 (Liu et al., 2010). Subsequently, experts proposed the concept of a broad food perspective and further expanded the extension of “food”, arguing that the purpose of food intake is not only to meet the needs of food, but also to meet the growing demand for health and nutrition. The development of the functional nutritional rice industry is also an objective requirement for practicing the broad food perspective (Liu et al., 2024).

Approximately more than 50% of the world’s residents consume rice as their staple food (Maclean et al., 2003). As a staple food crop, rice provides nutritional and health benefits to humans. Functional rice is bound to play a crucial role in guiding people from “being satisfied with having enough to eat and eating well” to “eating healthy and achieving health through eating”. Therefore, rice breeding should not only aim at high yield and high levels of pest and disease resistance (appearance and processing quality), but also pay attention to nutritional quality and functionality to meet the consumption demand for nutritious and healthy food. Theoretical and applied research on functional nutritional rice has increased to adapt to the changes in the consumption structure.

## 2 Definition of functional nutritional rice

Functional nutritional rice refers to a type of rice product that contains certain biologically active substances or special components in the rice cortex, embryo, and endosperm at higher or lower levels than ordinary rice that are beneficial to human health. Rice products or the biologically active substances or special components extracted through deep processing can balance nutrition in the human body after consumption and promote disease recovery, which conforms to the safety standards of rice. Furthermore, functional nutritional rice refers to rice products that are not used to treat diseases, suitable for consumption by the general population, and have a certain regulatory effect on human body functions (Liu et al., 2010). In addition to the seven nutrients essential for human growth and development, such as proteins, fats, carbohydrates, vitamins, minerals, water, and cellulose, functional nutritional rice contains certain special components that regulate and balance human physiological functions (Jin and Nie, 2023). Functional nutritional rice can enhance physiological defense mechanisms, prevent specific diseases, delay aging, control physical and mental conditions, among others (Wu et al., 2022). With the continuous breakthroughs in breeding technology and continuous improvement of living standards, more functional nutritional rice will appear on people’s dining tables in the future.

## 3 Nutritional composition of rice

### 3.1 Organizational structure of rice

The rice grain can be divided into two major parts: glume (rice husk) and caryopsis (brown rice). Rice glume, including the inner glume, outer glume, lodicule, and tip of the glume (elongated to form an awn), consists of four parts. The fruit obtained after removing the inner and outer rice glumes is the caryopsis (i.e., brown rice). The caryopsis is composed of three parts: cortex, endosperm, and embryo (Dou et al., 2018). The weight proportion of each rice component and brown rice grains varies considerably due to the different types and varieties of rice, soil, climate, and cultivation techniques. As the protective tissue of the endosperm, the rice husk accounts for 18–20% of the rice, has high contents of crude fiber and combined minerals (silicon), and a hard texture. The pericarp and testa account for 1.2–1.5% of the rice grain, the aleurone layer accounts for 4–6% of the rice grain, the endosperm accounts for 66–70% of the rice grain, and the embryo accounts for 2–3.5% of the rice grain (Wu et al., 2016).

### 3.2 Nutritional components of rice

Rice mainly contains carbohydrates, moisture, proteins, lipids, minerals, and vitamins (Safdar et al., 2023). The nutritional components of polished rice are far less than those of brown rice (brown rice is obtained after removing the rice husk). Functional components of rice are divided into nine major categories, including functional proteins (low-content glutelin, 26-kD anti-allergic protein), active polysaccharides (resistant starch, dietary fiber, and its analogs), functional oils (rich in unsaturated fatty acids), functional vitamins (β-carotene), essential trace elements (iron, zinc, and selenium), functional flavonoids, free radical scavengers, functional peptides, and essential amino acids for the human body (Su et al., 2007). The exploration and utilization potential of functional rice components is huge. For example, functional rice components can enhance human body functions and metabolic balance, thereby increasing the added value of rice and expanding the utilization range of rice. This makes rice not only food for human beings, but also an important raw material for new functional health products and the food industry.

### 3.3 Classification of functional nutritional rice

The classification of functional nutritional rice is diverse, and each classification method provides a mechanism of understanding, creating, and applying functional nutritional rice more extensively (Jin and Nie, 2023). With the constant advancement of scientific research technology and people’s higher pursuit of a healthy diet, the classification and application of functional nutritional rice is anticipated be more in-depth and extensive. This article summarizes the existing common types of functional nutritional rice, and the corresponding varieties and germplasm information list (**Table 1**) (Hu and Hu, 2021; Liu et al., 2024).

**Table 1:** Classification, cultivars, and germplasm of common functional nutritional. rice.

Classification		Function	Variety	Reference
Low Glutelin Rice		Assist in the treatment of kidney disease and diabetes	NM67, LGC-1、LGC-Katsu、LGC-Jun、LGC-soft	(Lida et al., 1993; Nishimura et al., 2005)
			Swarna	(Rathinasabapathi et al., 2015)
			Siddi、Sheetal	(Kamalaja et al., 2017)
			Doongara	(Williams et al., 2005)
			Cisokan, Batang Lembang, Margasari, Martapura, Tenggulang	(Indrasari et al., 2010)
			W3660、W0868	(Wan et al., 2004; Chen et al., 2023)
			Low Glutelin No.1, Wuyujing 2812, Chunyang, LGC-jun	(Kusaba et al., 2003; Wu et al., 2011)
High Resistant Starch Rice		Assist in reducing blood sugar and blood lipid, preventing colon cancer, and controlling weight	Zhefu 201	(Shen et al., 2006)
			Zhefu 111, Yitangxian No.1, Yitangjing No.1, Zhe da Liang you Yi tang xian No.1	(Wu et al., 2022)
			Jiangtangdao No.1, Youtangdao No.2, Yitang No.1, Gongmi No.3	(Hu and Hu, 2021; Yang et al., 2021; Du et al., 2023)
Giant Embryo Rice	Rich in γ-aminobutyric acid rice	Lower blood pressure, anti-anxiety, improve liver function, prevent cardiovascular and cerebrovascular diseases	Giant Embryo No. 1	(Pang, 1997)
			Haiminori, Beihai 269, Aoyu 359	(Maeda et al., 2001; Hu and Hu, 2021)
			Giant Embryo Red Japonica No.1, Ganju No.1, “Black Pearl”	(Hu and Hu, 2021)
			Liantang Giant Embryo Red No.1	(Hu and Hu, 2021)
			Giant Embryo Rice 1575, Giant Embryo Fragrant Glutinous 1547	(Fang et al., 2021)
			W025	(Hu and Hu, 2021)
Micronutrient-Rich Rice	Iron-rich rice	Prevent iron deficiency anemia	GCN4, Xi 026, Longqing No.4, Aixuenuo	(Hu and Hu, 2021)
	Zinc-rich rice	Improve immunity, promote growth and development, reproductive health	Yanghe Baipi Rice, Dalidao, Zheda Zinc Rice	(Hu and Hu, 2021; Wu et al., 2022)
	High calcium rice	Prevent calcium deficiency	Gongmi No.1	(Lucca et al., 2001a)
Colored Rice	Rich in anthocyanin rice	Antioxidant, prevent cardiovascular disease, cancer, protect eyesight	Minzixiang No.1, Ningnong Heijing, Minzixiang No.2, Dianxiangzi No.1	(Du et al., 2023)
			Zijing Rice	(Rahman et al., 2013)
	Rich in β-carotene rice	Improve immunity, prevent cataracts, cardiovascular disease	Golden Rice Generation 1	(Beyer, 2010)
			Golden Rice Generation 2	(Ye et al., 2000)
	Rich in astaxanthin rice	Antioxidant, prevent cardiovascular disease, cancer	Chijing Rice	(Higuera-Ciapara et al., 2006)
Other Functional Rice	Folic acid-rich rice	Prevent physiological metabolism, cardiovascular disease, neurodegenerative and other diseases and cancer	Chaoyangzao 18, Teqing, Dabai Gu 13	(Paine et al., 2005; Hu and Hu, 2021; Liu et al., 2024)
	Blood pressure-lowering short peptide-rich rice	Lower blood pressure	Kita-ACEI	(Hu and Hu, 2021; Liu et al., 2024)
	Hypoglycemic short peptide-rich rice	Lower blood sugar and blood lipid	Longdao 5-mGLP	(Hu and Hu, 2021; Liu et al., 2024)
	Rich in amino acids	Promote metabolic function	Ri-17、35R-17	(Gillies et al., 2008)
	Low Phytic Acid Rice	reduced levels of phytic acid in the grains	Lpa-1	(Yoshida et al., 1999)

#### 3.3.1 Low glutelin rice

Low glutelin rice refers to rice with a low glutelin content (< 4%) and a corresponding high prolamin content (Panlasigui and Thompson, 2006). Replacing ordinary rice with low glutelin rice can reduce creatinine levels, delay the progression of kidney disease, and improve intestinal function. Low glutelin rice is beneficial for patients with chronic kidney disease (Kusaba et al., 2003). Presently, the varieties of low glutelin rice are becoming increasingly abundant. Countries such as India, Indonesia, Bangladesh, Sri Lanka, Malaysia, and Australia, and international organizations, such as the International Rice Research Institute (IRRI) have extensively screened LGC rice varieties (**Table 1**). The LGC rice varieties that have been reported in India include Swarna (Rathinasabapathi et al., 2015), Siddi, and Sheetal (Kamalaja et al., 2017). The Australian variety is Doongara (Williams et al., 2005) and Indian varieties in Indonesia include Cisokan, Batang Lembang, Margasari and Martapura, and Tenggulang (Indrasari et al., 2010). LGC rice varieties in Bangladesh include BR-16, Pajam, and BR-3 (Howlander and Biswas, 2009). Japanese researchers obtained LGC-1 with a 4% decrease in glutelin content by chemical mutagenesis (Lida et al., 1993). China also attaches great importance to the selection and breeding of low glutelin content rice germplasm, and has cultivated new low glutelin rice varieties, such as W3660, W204, W0868, and W088 (Wan et al., 2004; Chen et al., 2023); new low-glutelin indica rice variety N198, Low Glutelin No.1, Wu 2812, Chunyang, LGC-Jun, and Yishenxiangsimiao (Wu et al., 2011; Chen et al., 2023; Liu et al., 2024).

#### 3.3.2 High resistant starch rice

Resistant starch is a new type of dietary fiber that cannot be enzymatically hydrolyzed in the small intestine and can only be degraded by microorganisms in the large intestine (Yang et al., 2021). Studies have confirmed that resistant starch causes a low insulin response and relatively stable blood sugar fluctuations (Sajilata et al., 2006). In addition, resistant starch can increase defecation, reduce constipation, reduce blood lipid content, and exert a weight loss effect to a certain extent (Kendall et al., 2004). The International Rice Research Institute developed Amylose Extender(AE) mutant rice that is rich in resistant starch and has a resistant starch content of 8.25% (Nishi et al., 2001). In 2013, South Korea developed a high-amylose rice mutant Goamy2 with a resistant starch content of 13.69% (Butardo et al., 2012). China has made considerable progress in the selection and research of high resistant starch rice, in addition to cultivating several germplasms and rice lines with high resistant starch content (**Table 1**), such as Zhefu 201 (Shen et al., 2006) and mutant RS111 (Yang et al., 2005). “Jiangtangdao No.1” is the first high resistant starch japonica rice variety (Shi et al., 2014) and the resistant starch content of Gongmi No.3 rice is greater than 10% (Luo et al., 2014). Other rice varieties rich in resistant starch include Ningnongheijing, Youtangdao No.2, and Yitangdao No.1 (Yang et al., 2021; Du et al., 2023).

#### 3.3.3 Giant embryo rice

Giant embryo rice is a special type of rice with substantially increased embryo volume and embryo weight, and is rich in γ-aminobutyric acid, γ-oryzanol, phenols, and minerals (Fang et al., 2021). Japan developed Haiminori as the world’s first giant embryo rice (**Table 1**) (Maeda et al., 2001). China has conducted research on the cultivation of giant embryo rice varieties (**Table 1**) and developed the giant embryo variety Giant Embryo No. 1 through hybridization (Pang, 1997), W025 (Liu et al., 2005). The existing giant embryo rice varieties include Giant Embryo No. 1, W025, Beihai 269, Aoyu 359, Hokuriku Glutinous 167, Chinese Glutinous 167, Giant Embryo Fragrant Glutinous 1547, Giant Embryo Red Japonica No. 1, Liantang Giant Embryo Red, and Giant Embryo 813B (Hu and Hu, 2021).

#### 3.3.4 Micronutrient-rich rice

Micronutrient-rich rice refers to rice varieties enhanced with essential trace elements such as iron, zinc, and selenium. These micronutrients are crucial for human health, and their deficiencies can lead to severe health issues like anemia and impaired immune function. Recent biofortification efforts have focused on increasing the content of these micronutrients in rice grains (Liu et al., 2021). Iron-rich rice varieties have been developed to combat iron deficiency anemia. For instance, Goto et al. (1999) developed transgenic rice rich in ferritin (Goto et al., 1999). Senguttuvel et al. (2023) provided a comprehensive review of biofortification strategies to improve iron and zinc content in rice (Senguttuvel et al., 2023). Their work highlights advances in conventional breeding, transgenic approaches, and genome editing techniques that have successfully increased micronutrient levels in rice grains (Lucca et al., 2001a). Similarly, zinc-rich rice varieties aim to improve immunity and promote growth and development. Studies have shown that biofortified rice with higher zinc content can significantly contribute to the dietary zinc intake in populations relying on rice as a staple food (Sedeek et al., 2023).

#### 3.3.5 Colored rice

Colored rice is characterized by the accumulation of anthocyanins in the seed coat of rice grains, which make the color of brown rice different from that of ordinary white rice. Due to varying anthocyanin contents, the seed coat of colored brown rice presents different colors, such as purple (black), red, yellow, and green. Purple (black) and red rice are the most common and no varieties with colored endosperm have been found in their natural state so far (Tong et al., 2011). According to statistics, China’s colored rice variety resources account for 90% of the world’s total and the remaining 10% of colored rice varieties are predominantly distributed in India, Bangladesh, Indonesia, Japan, Vietnam, the Philippines, and other East Asia countries (Zheng et al., 2021). Excellent colored rice varieties largely include ASD17 in India, A-201 in the United States and Chikubeniwamochi in Japan [ (Jaksomsak et al., 2020), **Table 1**]. Furthermore, with the development of local tourism agriculture, colored rice has become well-known among local populations as ornamental rice. Colored rice not only has ornamental functions, but can also be consumed and ultimately promote the development of agriculture and increase the income of farmers.

#### 3.3.6 Other functional rice varieties

Genetic engineering technology and CRISPR/CaS9 gene editing technology have largely been used to improve and increase the contents of some elements, such as iron, zinc, vitamins, flavonoids, and carotenoids in rice (Beyer et al., 2002). Currently, the first and second generations of Golden Rice with substantially high β-carotene content (Ye et al., 2000; Paine et al., 2005; Beyer, 2010) and nutritious rice with high folic acid content have been successfully bred (Storozhenko et al., 2007). Introduction of the wheat gene *HPPK/DHPS* into rice has been shown to increase vitamin B9 (folic acid) content of rice by 75% (Gillies et al., 2008). Rice varieties Ri-17 and 35R-17 that are rich in amino acids essential for human metabolism have also been developed and cultivated (Gillies et al., 2008). Rice varieties, such as Ningjing 1-FAD3 is rich in α-linolenic acid and Longdao 5-mGLP variety is rich in hypoglycemic peptides that can reduce blood sugar and blood lipids (Du et al., 2023).

#### 3.3.7 Low phytic acid rice

Low phytic acid rice refers to rice varieties that have reduced levels of phytic acid in the grains. Phytic acid is known to bind essential minerals like iron, zinc, and calcium, forming insoluble complexes that inhibit their absorption in the human digestive system (Yoshida et al., 1999). By reducing phytic acid content, low phytic acid rice enhances the bioavailability of these essential minerals, contributing to better nutritional status. Additionally, lower phytic acid levels can reduce environmental phosphorus pollution since phytic acid is a primary storage form of phosphorus in seeds. Examples of low phytic acid rice varieties include ‘Lpa-1’ (Rutger et al., 2004).

## 4 Genes associated with functional nutritional rice improvement

Biofortification of staple crops to increase the intrinsic functional and nutritional components in the edible parts is considered one of the most economical and sustainable methods to overcome “hidden hunger” (Long et al., 2013). However, limited genetic diversity exists among rice varieties with certain nutritional components (such as resistant starch, iron, and zinc, resulting in fewer genetic resources. Additionally, rice lacks the metabolic pathways associated with the synthesis of certain functional components (e.g., astaxanthin and β-carotene), thereby posing a challenge to breeding functional nutritional rice varieties that meet human nutritional needs using conventional breeding methods. Therefore, utilizing genetic engineering to alter the metabolic pathways in rice as well as enrich functional and nutritional components in the rice endosperm is a crucial approach for functional and nutritional enhancement. The metabolic pathways used for functional and nutritional enhancement, and the genes applied to such enhancement are summarized in **Table 2**.

**Table 2:** List of genes associated with functional nutritional rice improvement.

Genes	Description	Function	Reference
Starch synthesis			
*SSIIIa*	Soluble starch synthase	Mutation of *SSIIIa* increases RS content	(Yadav et al., 2010)
*SSIIIb*	Soluble starch synthase	Mutation of *SSIIIb* doesn’t increase RS content; Mutation both *SSIIIa* and *SSIIIb* increased RS content observably	(Zhou et al., 2016; Huang et al., 2024)
*SBEI*	Starch branching enyzme	Mutation or RNAi of *SBEI* increases RS content	(Wei et al., 2010; Tsuiki et al., 2016; Guo et al., 2020; Chen et al., 2022; Wang et al., 2023)
*SBEIIb*	Starch branching enyzme	Mutation or RNAi of *SBEIIb* increases RS content	(Wei et al., 2010; Tsuiki et al., 2016; Guo et al., 2020; Chen et al., 2022; Wang et al., 2023)
Secondary metabolites Synthesis			
*Rc* and *Rd*	proanthocyanidins	Seed coat appears red when both *Rc* and *Rd* are present; appears brown when only *Rc* is present; appears white when *Rc* is absent	(Finocchiaro et al., 2010)
*Pb* and *Pp*	Anthocyanins	Seed coat appears white when *Pb* is absent; appears brown when only *Pb* is present; purple when both *Pb* and *Pp* are present	(Furukawa et al., 2007)
*MYB*, *bHLH*, *HY5*, and *WD40*	Anthocyanins	Overexpression these genes increases anthocyanin content in the endosperm	(Zhu et al., 2009)
*sZmPSY1*、*sPaCrtI*、*sCrBKT*、*sHpBHY*	astaxanthin	Overexpression these genes increases astaxanthin content in the endosperm	(Zhu et al., 2017)
*psy*, *crtI*, *lcy*	β-carotene	Overexpression these genes increases β-carotene content in the endosperm	(Ye et al., 2000; Beyer, 2010)
Embryonic development			
*GE*	Giant embryo	Mutation of *GE* results in an enlarged embryo	(Yang et al., 2013; Zhu et al., 2018)
*LE*	LARGE EMBRYO	Mutation of *GE* results in an enlarged embryo	(Taramino et al., 2003)
*PLA3*/*GO*	Glutamate carboxypeptidase	Mutation of *GE* results in an enlarged embryo	(Nagasawa et al., 2013)
Protein synthesis			
*LGC-1*	Low glutelin content 1	Mutation of *LGC-1* reduces the glutelin content in grains	(Lida et al., 1993)
*OsAAP6*	Amino acid permease; Amino acid transporter	Mutation of *OsAAP6* reduces the glutelin content in grains	(Miyahara, 1999; Longchamp et al., 2015)
*OsAAP10*	Amino acid transporter	Mutation of *OsAAP10* reduces the glutelin content in grains	(Longchamp et al., 2015)
*OsGluA1、OsGluA2、OsGluA3、OsGluB2、OsGluB6、OsGluB7、OsGluC、OsGluD*	Glutelin genes	Mutation several of these genes reduce the glutelin content in grains	(Takahashi et al., 2019)
*OsGluA1*, *OsGluA2*, *OsGluA3*, *OsGluB1a*, *OsGluB1b*, *OsGluB2*, *OsGluB4*, *OsGluB5*	Glutelin genes	Mutation several of these genes reduce the glutelin content in grains	(Yang et al., 2022)
Absorption and transport of mineral elements			
*OsIRT1*	Iron-regulated metal transporter	Overexpression of *OsIRT1* increases Fe and Zn concentration in grains	(Miller, 2013)
*AtIRT1*	Iron-regulated metal transporter	Overexpression of *AtIRT1* increases Fe and Zn concentration in grains	(Lee et al., 2009a)
*OsYSL15*	Iron(III)-deoxymugineic acid transporter	Overexpression of *OsYSL15* increases Fe concentration in grains	(Lee and An, 2009)
*OsNAS1*	Nicotianamine synthase	Overexpression of *OsNAS1* increases Fe and Zn concentration in grains	(Lee et al., 2009b)
*OsNAS2*	Nicotianamine synthase	Overexpression of *OsNAS2* increases Fe and Zn concentration in grains	(Lee and An, 2009; Lee et al., 2009a; Lee et al., 2012)
*OsNAS3*	Nicotianamine synthase	Overexpression of *OsNAS3* increases Fe and Zn concentration in grains	(Boonyaves et al., 2016)
*HvNAS1*	Nicotianamine synthase	Overexpression of *HvNAS1* increases Fe and Zn concentration in grains	(Lee et al., 2011)
*GmFER*	Ferritin gene	Overexpression of *GmFER* increases Fe and Zn concentration in grains	(Goto et al., 1999)
*PvFER*	Ferritin gene	Overexpression of *PvFER* increases Fe concentration in grains	(Senguttuvel et al., 2023)
*AtFRD3*	Ferric reductase defective*;* Citrate transporter	Overexpression of *AtFRD3* increases Fe and Zn concentration in grains	(Durrett et al., 2007)
*OsYSL2*	Metal-nicotianamine transporter	Overexpression of *OsYSL2* increases Fe and Zn concentration in grains	(Ishimaru et al., 2010)
*OsVIT2*	Vacuolar membrane transporter	Mutation of *OsVIT2* increases Fe and Zn concentration in grains	(Bashir et al., 2013)
*OsYSL9*	Fe(III)-DMA and Fe(II)-nicotianamine; (NA) influx transporter	Mutation of *OsYSL9* increases Fe concentration in grains	(Senoura et al., 2017)
*OsIRO2*	bHLH protein; *IRON‐RELATED BHLH TRANSCRIPTION FACTOR 2*	Overexpression of *OsIRO2* increases Fe and Zn concentration in grains	(Ogo et al., 2011)
*NRT1.1b*	Nitrate-transporter gene	Overexpression of *NRT1.1b* increases Fe and Zn concentration in grains	(Zhang et al., 2019)
*OsASTOL1*/*OsRCS1*	Cysteine synthase	Mutation of *OsASTOL1* at 566^th^ nucleotide from G to A increases Se concentration in grains	(Sun et al., 2021)

### 4.1 Genes involved in the starch synthesis pathway

Starch synthesis in the endosperm begins with the photosynthetic production of glucose, which is then transported into plastids by transporter proteins and converted into ADP-glucose by the enzyme ADP-glucose pyrophosphorylase (Kammerer et al., 1998; Bouis et al., 2011). ADP-glucose is used to synthesize amylose by granule-bound starch synthase and amylopectin synthesized by the coordinated action of soluble starch synthase, starch branching enzyme (SBE/BE), and starch debranching enzyme (Smith, 2008). Regular rice has a substantially low resistant starch content of <1%. Increasing resistant starch content in rice can effectively reduce starch digestion rate, increase satiety, and prevent various diseases, such as diabetes and obesity (Ballicora et al., 2003). This is a crucial direction for rice quality improvement (Sharma et al., 2008).

The formation of resistant starch in rice is regulated by the soluble starch synthase gene *SSIIIa* and granule-bound starch synthase I (Waxy). Knocking out *SSIIIa* promotes accumulation of resistant starch in rice (Yadav et al., 2010). Knocking out *SSIIIa* (*SS3a*) and *SSIIIb* (*SS3b*) genes in *Nipponbare* increased resistant starch content in *SS3a* mutants from 0.58% in the wild-type rice to approximately 5%; however, no significant differences were observed in the resistant starch content of *SS3b* mutants. However, resistant starch content in the double mutants (*ss3a ss3b*) further increased to 9.5% (Zhou et al., 2016). Editing *SS3a* and *SS3b* genes in Zhonghua 11 has also yielded similar results (Huang et al., 2024). The absence of *SBEI* and *SBEIIb* genes in rice results in increased resistant starch accumulation (Tsuiki et al., 2016; Guo et al., 2020; Wang et al., 2023). Inhibiting the expression of SBE encoding genes *SBEI* and *SBEIIb* increases resistant starch content in rice to 14.9% (Wei et al., 2010; Chen et al., 2022). Miura et al. crossed natural mutants of *SBEI* and *SBEIIb* to develop a high-resistant double mutant rice (*seb1 seb2b*), which had a resistant starch content of 35.1% in the endosperm (Zhu et al., 2012; Miura et al., 2021).

### 4.2 Genes involved in secondary metabolite synthesis

The accumulation of anthocyanins/proanthocyanidins in the seed coat confers brown rice its distinctive color and certain nutritional benefits (Xu et al., 2015). Red rice contains only proanthocyanidins, while black and purple rice contain both anthocyanins and proanthocyanidins (Finocchiaro et al., 2010). The red seed coat of rice is controlled by the complementary interaction of two major genes, *Rc* and *Rd*. The rice seed coat appears red when both *Rc* and *Rd* are present; the seed coat appears brown when only *Rc* is present; and the seed coat appears white when *Rc* is mutated to *rc* (Furukawa et al., 2007). The purple seed coat of rice is regulated by two dominant complementary genes, *Pb* and *Pp*. The rice seed coat is white when *Pb* is absent; the seed coat is brown when only *Pb* is present; and the seed coat is purple when both *Pb* and *Pp* are present (Rahman et al., 2013). Nutritionally enhanced rice germplasm has been developed using endosperm-specific promoters to introduce metabolic pathways of secondary metabolites, such as astaxanthin, anthocyanins, and β-carotene into rice. These genetic introductions result in the display of various colors, such as red, purple, and yellow by rice seeds. Astaxanthin is biosynthesized from β-carotene by β-carotene ketolase (BKT) and β-carotene hydroxylase (BHY) (Higuera-Ciapara et al., 2006). The initial precursors of carotenoids (including β-carotene) are synthesized via the 2-C-methyl-D-erythritol-4-phosphate pathway (Zhu et al., 2009). Biofortified rice germplasm with high anthocyanin content in the endosperm has been developed by simultaneously transforming rice with genes, such as *MYB*, *bHLH*, *HY5*, and *WD40*. Rice with endosperm rich in astaxanthin has been successfully developed (Zhu et al., 2009) by introducing genes, such as *sZmPSY1*, *sPaCrtI*, *sCrBKT*, and *sHpBHY*. The first generation of Golden Rice with β-carotene-rich endosperm was developed (Zhu et al., 2017) by introducing the phytoene synthase (*PSY*) gene from daffodil, lycopene β-cyclase (*LCY*) gene, and carotene desaturase (*CRTI*) gene from bacteria into rice (Beyer, 2010). Subsequent modifications by introducing the *PSY* gene from maize and *CRTI* gene from bacteria into rice further increased β-carotene content by nearly 23-fold (Ye et al., 2000).

### 4.3 Genes related to embryo development

The embryo of giant embryo rice is two to three times larger than that of regular rice and several genes regulating rice embryo size have been identified. The giant embryo (*GE*) gene was the first to be identified in rice (Zhu et al., 2018). *GE* in rice encodes a P450 protein that is a member of the CYP78 family. The indole-3-acetic acid content in the rice grain of *ge* mutants reduced considerably, thereby affecting the expression of genes involved in the cell cycle. Loss of *GE* function results in an enlarged embryo and a reduced endosperm (Yang et al., 2013). Other than *GE* cloning, mutations in the *Goliath* (*GO*), *PLASTOCHRON3* (*PLA3*), and *LARGE EMBRYO* (*LE*) genes have resulted in enlarged embryos, with *PLA3* and *GO* being the same gene. *PLA3*/*GO* encodes a glutamate carboxypeptidase II, which is a member of the M28 family of metalloproteases (Nagasawa et al., 2013). *LE* is the first C3HC4-type RING finger protein reported to be involved in embryo morphogenesis. Inhibition of *LE* gene expression by RNAi technology results in an enlarged rice embryo, although the specific molecular regulatory mechanism remains unclear (Taramino et al., 2003).

### 4.4 Genes related to protein synthesis

Protein is the second largest storage substance in rice endosperm, predominantly consisting of four components: glutelin, prolamin, globulin, and albumin. Glutelin has the highest content, accounting for approximately 60–80% of the storage proteins (Lee et al., 2019). The eating quality of rice varies with similar amylose content and a low protein content enhances the eating quality of rice. Therefore, reducing protein content is an effective strategy for improving the eating quality of rice (Saito et al., 2012; Yang et al., 2019). Studies have revealed that mutations in the *LGC1* gene on chromosome 2 of rice can reduce glutelin content in grains to less than 4% (Miyahara, 1999). Amino acid transporter encoding genes *OsAAP6* and *OsAAP10* have been edited using CRISPR/Cas9 technology, and mutations in *OsAAP6* and *OsAAP10* substantially reduce rice protein content and improve taste scores (Miyahara, 1999; Longchamp et al., 2015). Rice glutelin synthesis genes are divided into four subfamilies: *GluA*, *GluB*, *GluC*, and *GluD*, which are directly related to rice protein content and taste quality (Takahashi et al., 2019; Wang et al., 2020). Simultaneous knocking out of eight highly expressed glutelin family genes (*OsGluA1*, *OsGluA2*, *OsGluA3*, *OsGluB2*, *OsGluB6*, *OsGluB7*, *OsGluC*, and *OsGluD*) using CRISPR/Cas9 technology resulted in seven different mutant combinations. Consequently, the protein content of rice decreased to varying degrees, and the hardness, appearance, and taste value of rice improved considerably (Takahashi et al., 2019). New rice germplasm with reduced glutelin content has been developed using CRISPR/Cas9 technology by knocking out multiple glutelin encoding genes (*OsGluA1*, *OsGluA2*, *OsGluA3*, *OsGluB1a*, *OsGluB1b*, *OsGluB2*, *OsGluB4*, and *OsGluB5*) (Yang et al., 2022).

### 4.5 Genes related to mineral element absorption and transport pathways

Over a billion people worldwide have iron and zinc deficiencies. Enriching rice grains with iron and zinc is considered the most effective method of addressing these nutritional deficiencies (Wessells and Brown, 2012; Chen et al., 2022). The pathways of iron and zinc absorption, transport, and storage in rice have been extensively studied. Biofortification can be achieved through various methods, such as increasing the binding of iron/zinc with phytosiderophores and plant iron carriers at the root zone, in turn, enhancing iron/zinc absorption, promoting long-distance transport of iron/zinc within the plant, increasing endosperm-specific storage of iron and zinc, and altering the expression of transcription factors (**Table 2**).

The iron-regulated metal transporter 1 (*IRT1*) gene encodes iron (II) and zinc (II) transporters in plant roots. Overexpression of *OsIRT1* or *AtIRT1* in rice substantially increases the accumulation of iron and zinc in rice grains (Lee and An, 2009; Miller, 2013). The yellow stripe 1-like gene 15 (*OsYSL15*) is associated with the uptake of Fe(III)-DMA. Similarly, overexpression of *OsYSL15* increases the iron content in mature rice seeds by approximately 1.2-fold (Lee et al., 2009a). Phytosiderophores and nicotianamine are chelators involved in the internal transport of metals in plants. These chelators are crucial for iron absorption in rice and possibly facilitate zinc absorption. Studies have shown that overexpression of nicotianamine synthase genes *OsNAS1*, *OsNAS2*, and *OsNAS3* in rice considerably increases iron and zinc contents in rice grains (Lee et al., 2009b; Lee et al., 2011; Lee et al., 2012; Boonyaves et al., 2016). Similarly, introducing the barley gene *HvNAS1* into rice or simultaneously transferring several different *NAS* genes from rice and barley into rice increases iron and zinc contents in rice (Masuda et al., 2009; Johnson et al., 2011). Specific expression of the ferritin-encoding gene in the rice endosperm is an effective strategy for increasing iron content in rice grains (Lucca et al., 2001b; Banakar et al., 2017). Regulating the expression of soybean *FER* (*GmFER*) or common bean *FER* (*PvFER*) genes through endosperm-specific promoters substantially increases endosperm iron content (Goto et al., 1999; Banakar et al., 2017). Arabidopsis FERRIC REDUCTASE DETECTIVE 3 (*AtFRD3*) is a transporter that loads citrate into the xylem of root columns, thereby increasing iron mobility within plants (Durrett et al., 2007). Iron and zinc contents increased by 5.4- and 2.4-fold, respectively in the polished grains of transgenic rice lines with constitutive expression of *AtFRD3* when compared to those of control plants (Wu et al., 2018a). Additionally, *pOsSUT1* plants with polished grain iron content 4.4-fold higher than those of wild-type plants have been developed through the expression of the iron(II)-NA transporter encoding gene *OsYSL2* under the control of the rice *SUCROSE TRANSPORTER1* promoter (*pOsSUT1*) (Ishimaru et al., 2010). Vacuolar membrane transporter 2 (*OsVIT2*) encodes an iron and zinc transporter on the vacuolar membrane. Knocking out *OsVIT2* reduces the accumulation of iron and zinc in the flag leaves and increases their transport to the sink tissues, which directly leads to high iron and zinc contents in rice seeds (Bashir et al., 2013). Studies have shown that *OsYSL9* can transport Fe-NA/DMA complexes from the endosperm to the embryo during seed development. Knocking down *OsYSL9* increases the endosperm iron content by more than twofold (Senoura et al., 2017). The iron content of brown rice increased by more than twofold through overexpression of the transcription factor *IRON‐RELATED BHLH TRANSCRIPTION FACTOR 2* (*OsIRO2*), which is involved in iron deficiency responses in rice (Ogo et al., 2011). Introducing multiple genes simultaneously can produce stronger biofortification effects than single gene transformations that produce weak biofortification effects. For example, introducing *GmFER*, *HvNAS1*, and *OsYSL2* simultaneously increases iron and zinc contents in polished rice by 6- and 1.6-fold, respectively (Ogo et al., 2011). Introducing *OsNAS2* and *GmFER* into rice increases iron and zinc contents in polished rice by 7.5- and 3.5-fold, respectively (Masuda et al., 2012). Simultaneous introduction of *AtFRD3*, *AtNAS1*, and *PvFER* increases iron and zinc contents in polished rice by 5.4- and 2.4-fold, respectively (Durrett et al., 2007).

Selenium is an essential microelement for humans and its deficiency can lead to potential health risks. Studies have shown that different genotypes of the same plant species exhibit marked differences in selenium absorption. Selenium and sulfur belong to the same group, and have similar chemical structures and properties, suggesting that both elements can enter plants through the same metabolic pathway (Trijatmiko et al., 2016). Numerous proteins involved in selenium absorption and transport in rice have been reported. For example, SULTR1;1 and SULTR1;2, which are potential sulfate transporters, thereby facilitating sulfur absorption (Sun et al., 2021). Furthermore, overexpression and knockout of *OsPT2* in plants considerably increases and decreases selenite uptake, respectively, indicating that *OsPT2* is involved in selenite uptake (Zhang et al., 2014). Overexpression of *OsPT8* substantially increases selenium content in tobacco plants, indicating that *OsPT8* is also involved in selenite absorption and transport (Song et al., 2017). Yeast expressing *OsNIP2;1* enhances selenite uptake at pH 3.5 and 5.5, but not at pH 7.5 (Zhao et al., 2010). Overexpression of *NRT1.1b* considerably increases selenium content in both rice stems and grains (Zhang et al., 2019). A mutation of the 566^th^ base of the *OsASTOL1* gene from G to A in rice resulted in the change of the 189^th^ amino acid from serine to aspartate, in turn, producing the *OsASTOL1^S189N^* gain-of-function mutant. This mutant not only reduces arsenic accumulation in rice grains but also increases selenium content by more than onefold (Zhang et al., 2014).

In conclusion, with the advancement of biotechnology and in-depth study of functional genes, numerous gene functions have been discovered and reported. The application of technologies, such as CRISPR/Cas9, RNAi, and gene overexpression to modify rice metabolic pathways and enrich nutrients in the rice endosperm is an effective approach for biofortification. However, the practical application of these technologies is associated with challenges, such as the safety assessment of gene-editing technologies, stability of nutrients in rice, and how to improve consumer acceptance.

## 5 Progress in functional nutritional rice breeding

### 5.1 Conventional breeding

Conventional breeding falls under the breeding 2.0 era, which is a breeding era characterized by experimental design, field statistics, and analysis (Wallace et al., 2018) Chinese rice breeder Huang Yaoxiang, who was internationally known as the “father of semi-dwarf rice”, developed the Guangchangai rice variety through hybrid breeding in 1959. Subsequently, other breeders bred dwarf varieties, such as Aijiaonante and Dee-geo-woo-gen. The dwarf rice variety is resistant to lodging and fertilizer, and has a high yield (Tu and Wang, 2019). Chinese breeders pioneered the first revolution in rice breeding and made substantial contributions to the first Green Revolution (Zheng et al., 2024). The IR8 developed by the IRRI in 1966 and was bred using Dee-geo-woo-gen, has become an internationally recognized landmark achievement in the “Green Revolution” and the first breakthrough in the history of rice breeding (Zheng et al., 2024). From 1962 to 1988, IRRI developed a total of 34 rice varieties, with their selection being achieved through conventional breeding. Most of the varieties have good appearance quality, high yield, high rice quality, and strong stress resistance, and they have been introduced to several countries globally for planting and promotion. The conventional breeding of functional nutritional rice, which is often achieved through hybridization and backcrossing based on the appearance phenotype, relies on the germplasm resources of functional rice. For example, colored rice such as black, purple, red, and green rice are easy to distinguish in appearance and can be directly selected for breeding with relatively high efficiency. In addition, even though several biofortified rice varieties for protein and zinc have been developed in Asia (Hu and Hu, 2021; Wu et al., 2022). the selection of fragrant rice germplasm is determined by sensory evaluation; that is, smelling and chewing. However, the disadvantage is that sensory evaluation is limited to rice varieties that are easily recognizable in appearance.

### 5.2 Combination of conventional breeding and marker-assisted breeding

In 1953, the discovery of the DNA double helix model marked the arrival of the era in which molecular biology (Watson and Crick, 2003) and genetics research was advanced to the molecular level and crop breeding was ushered into the era of molecular breeding. Molecular breeding, also known as breeding 3.0, is a combination of modern biotechnology and conventional breeding methods guided by theories, such as genetics and molecular biology, to achieve an organic combination of phenotype and genotype selection, and cultivate excellent new varieties (Wallace et al., 2018). In conventional plant breeding, genetic variation is primarily identified through observation and measurement. The development of molecular biology has enabled researchers to detect variations in DNA sequences that can affect phenotypic traits and DNA sequence variations can be detected using various techniques. Molecular marker technology, which confers the advantages of abundant quantity, site specificity, dominant or codominant, high resolution, and good stability (Jander et al., 2002), has gradually developed. Some biologically active substances or special components in rice cannot be identified through phenotypic observations. Based on the identification of key genes involved in the synthesis or metabolism of biologically active substances, molecular markers that are closely linked to such genes can be used to aggregate multiple traits into one variety. A combination of molecular markers and conventional breeding has the advantages of clear objectives, high accuracy, no environmental interference, and an accelerated breeding process (Jin and Nie, 2023). Although most genes regulating crucial agronomic traits have been identified in rice, few genes with nutritional value and unique biologically active ingredients have been identified (**Table 2**). Moreover, fewer genes can be used for molecular marker-assisted selection, which considerably limits the widespread application of functional rice breeding. At the same time, the play of some key regulatory genes requires a specific genomic background. Therefore, in addition to the selection of key genes, genome selection must be carried out.

### 5.3 Mutation breeding

Mutation breeding refers to the use of chemical, physical, and other factors to induce mutations in organisms under human conditions. Grain crops have regularly been the focus of mutagenesis breeding and according to statistics, the number of varieties bred through mutagenesis accounts for approximately 10% of the total number of varieties bred during the same period. For example, an early Chinese *Indica* rice variety called Yuanfengzao was developed using IR8 and 60Co radiation treatment, followed by mutagenesis selection (Zhang and Tai, 2001).

Functional nutritional rice can be obtained by mutagenesis screening and identification of specific functional nutritional components. For example, the world’s first giant embryo rice “Haiminori” (**Table 1**) was bred by crossing the giant embryo mutant EM40 that was selected by chemical mutagenesis of Kinmaze with the high-yielding variety Akenohoshi (**Table 1**). The embryo volume of the giant embryo rice is three- to fourfold that of ordinary rice (Maeda et al., 2001). Low glutelin content-1 (LGC-1) rice cultivar was developed through chemically-induced mutation of Nihonmasari, a high-quality rice cultivar from Japan to obtain a mutant NM67 with substantially reduced endosperm glutelin content. The mutant was then backcrossed with the recurrent parent Nihonmasari for the first time to obtain LGC-1 (Lida et al., 1993). Compared to conventional rice, LGC-1 rice has low glutelin content (<4%), increased prolamin content, and considerably reduced total absorbable protein, which can be consumed by patients with diabetes (Kusaba et al., 2003). *LGC1* is a key germplasm resource for breeding functional rice and is widely used by breeders. Low glutelin protein cultivars, such as LGC-Katsu and LGC-Jum, have been developed successively using LGC-1 as a parent (Nishimura et al., 2005).

The major challenge associated with mutagenesis breeding is the low frequency of beneficial mutations, as well as difficulties in orientation and nature of mutations. Therefore, it is essential to improve mutagenesis efficiency, rapidly identify and screen mutants, and explore targeted mutagenesis pathways. Mutation breeding should be combined with other breeding technologies to improve breeding efficiency. Wan Jianmin from Nanjing Agricultural University in China introduced the LGC-1 rice cultivar and its *LGC1* gene into the dominant rice varieties. Through hybridization, backcrossing, and multiple crossing, combined with phenotype identification, a broad range of low glutelin protein content rice varieties, such as W3660, W204, W1721, W0868, and W088 have been bred successively using molecular marker-assisted selection. The low glutelin protein content of rice has decreased considerably and globulin is deficient, thereby resulting in a further decrease in its absorbable protein content and postprandial blood glucose levels. The applicable population has further expanded, and commercial planting and promotion of this rice variety has begun (Wan et al., 2004; Chen et al., 2023).

### 5.4 Genetic engineering technology improvement

Genetic engineering technology was marked by the establishment of DNA recombination technology in the 1970s (Cohen et al., 1973) and was used in the 1980s for transgenic plants, also known as genetically modified (GM) plants. Genetic engineering technology refers to the use of genetic manipulation methods to develop plants with new superior and artificially-designed traits. GM breeding has been the fastest-developing field of agricultural biotechnology application since the 1980s. The cumulative planting area of GM crops worldwide between 1996 and 2019 was 2.7 × 10^9^ hectares (hm^2^) (Li et al., 2023). A GM tomato with delayed ripening (Flavr Savr) was approved for consumption on May 18, 1994. The Flavr Savr tomato was the first GM tomato approved for commercial cultivation by the Food and Drug Administration of the United States, and its acceptance among consumers was positive (Kramer and Redenbaugh, 1994). The accumulation of specific biologically active substances in the endosperm to enhance the nutritional quality of rice can be achieved by expressing and modifying key genes involved in the synthesis of biologically active substances in rice or restructuring the synthesis pathways of certain biologically active substances that cannot be directly synthesized in the endosperm. The endosperm of rice seeds is an ideal bioreactor for metabolic engineering and food bioenhancement (Zhu et al., 2017), such as the first transgenic of rice developed for high iron content by overexpression of *OsNAS1* (Lee et al., 2009b). Currently, various nutrient-enriched rice varieties have been developed based on these strategies. The common target micronutrients obtained through bioenhancement breeding are iron, zinc, vitamins, flavonoids, and carotenoids. Most of the genes related to carotenoid synthesis in the rice endosperm are either unexpressed or exhibit low expression. Anthocyanins and carotenoids have become popular research directions when compared to other bioactive compounds due to their distinct synthetic pathways.

Golden Rice is a new type of GM rice with three newly inserted coding genes, which express *PSY* from daffodil, *LCY*, and *CRTI* from the soil bacterium *Erwinia uredovora* that regulate β-carotene synthesis in the rice endosperm. Golden Rice is rich in carotene, which can be converted into preformed vitamin A in the human body (Ye et al., 2000; Beyer, 2010). Astaxanthin is a red-colored xanthophyll carotenoid that is essential for maintaining organism balance and reducing the accumulation of senescent cells. However, astaxanthin is only produced by some algae and yeast, and cannot be produced by most higher plants. Therefore, ways of obtaining astaxanthin, which has strong antioxidant properties, from the daily diet of humans are few. A research team led by Liu Yaoguang from South China Agricultural University in Guangzhou, Guangdong, China has developed a synthetic metabolic engineering strategy and a multigene stacking system TGSII using rice endosperm-specific promoters. The researchers combined astaxanthin with the common grain crop rice to develop “Purple Endosperm Rice” that has a high anthocyanin content (Zhu et al., 2017). Four key genes involved in carotenoid biosynthesis, namely phytoene synthase (*sZmPSY1*), phytoene desaturase (*sPACrTI*), β-carotene ketolase (*sCrBKT*), and β-carotene hydroxylase (*sHspBHY*) genes have been expressed in rice using the TGSII system (Zhu et al., 2017). Different combinations of carotenoid/ketocarotenoid/astaxanthin biosynthetic pathways were reconstructed in the rice endosperm, resulting in purple embryo rice with astaxanthin exhibiting high antioxidant activity and removal of the transgenic screening marker. The GM rice developed using this technology are Canthaxanthin and Astaxanthin rice. The study used exogenous genes to compensate for the endogenous astaxanthin synthesis pathway in rice, in turn, leading to astaxanthin synthesis in the rice endosperm for the first time (Zhu et al., 2018). Enhancing the expression of related transporter genes can improve the absorption and utilization efficiency of iron and zinc in plants, which is an effective transgenic strategy for increasing crop iron and zinc contents (Blancquaert et al., 2015). Moreover, the coexpression of lactoferrin (iron chelating glycoprotein) and ferritin genes increases iron content in crops (Goto et al., 1999; Drakakaki et al., 2000). Simultaneous expression of genes encoding ferritin and nicotianamine synthase in crops increases zinc and iron contents (Wirth et al., 2009). Numerous biofortified crops with high iron and zinc contents, such as rice, corn, and wheat (Kumar et al., 2019), have been developed using genetic engineering technology. Crops biofortified with nutrients have a great potential to address global mineral deficiencies (Jin and Nie, 2023; Liu et al., 2024).

### 5.5 Gene editing technology

Genome editing technology is a breakthrough technology in the field of biological genetic manipulation after transgenic technology. The CRISPR/Cas system is a novel genome editing technology that emerged after the zinc finger nuclease and transcription activator-like effect nuclease technologies. This technology involves site editing of specific genome sequences using a guide RNA and the cooperating nuclease Cas9, and has the advantages of simplicity and efficiency (Mahfouz et al., 2011; Jinek et al., 2012). The CRISPR/Cas9 system has enabled genome editing in various crops, such as rice, wheat, corn, soybean, rapeseed, tomato, watermelon, strawberry, and it is at the forefront of research in rice and wheat, thereby providing important technical support for crop variety improvement and new variety cultivation.

A japonica rice variety cultivated using LGC-1 as the donor parent was used as the transgenic recipient. The starch branching enzyme IIb (*SBEIIb*) gene was knocked out using CRISPR/Cas9 technology to obtain a high-resistance starch and low glutelin protein mutant line without transgenic components. The apparent starch, resistant starch, and glutelin contents of the mutant line increased by approximately 1.8-fold, 6%, and 2% (Tsuiki et al., 2016; Guo et al., 2020), respectively. Chen et al. (2022) used CRISPR/Cas9 technology to simultaneously edit nine glutelin genes in LGC germplasm and obtained nine homozygous edited strains without T-DNA (Chen et al., 2022). Due to different regulatory policies on gene editing in various countries, the cultivation of functional nutritional rice bred using CRISPR/Cas9 is still at the laboratory and publication stage. Solving specific regulatory challenges or consumer safety concerns is a complex problem. Due to length limitation and different focus, this review cannot discuss all the concerns you have raised. However, in the revised manuscript, we raised these questions to draw attention from all aspects.

From the perspective of cultivated varieties, the breeding of functional nutritional rice predominantly involves the aforementioned five aspects. However, currently, several countries in the world, including China, have regulations on the management of GM crops. Genetic engineering and gene editing products cannot be directly planted or consumed at this stage. In the actual breeding process of functional nutritional rice, multiple methods are often used in combination to accelerate the research and development process of functional rice. Although some progress has been made in molecular breeding, mutagenesis, genetic engineering, and gene editing technologies, the maturity of these technologies, especially in identifying and utilizing functional rice germplasm resources, still needs to be improved. Because the entire experimental process is still time-consuming and costly, it limits its widespread commercial application. The implementation of these technologies highly relies on advanced scientific instruments and relatively complex technical means, which limits the promotion of the technology. Due to the limited investment in research and development, the gap between developing countries and developed countries may last for 20-30 years. Moreover, research on the genes and molecular mechanisms related to functional nutritional rice is still very limited due to the pleiotropy of genes. Therefore, many unknown factors are still unpredictable when using these technologies for genetic improvement of functional nutritional rice. Especially in gene editing breeding, due to patent protection of core technologies, the discovery and identification of excellent genes in germplasm resources are lagging behind, which affects breeding efficiency and accuracy. In addition, due to the conservative safety evaluation and approval processes for genetically engineered rice in different countries, the commercialization process of genetically modified breeding has been slow.

## 6 Problems associated with and prospects of functional nutritional rice breeding

### 6.1 Problems

Although rice breeding ranks first globally when compared to other crops, the primary goals of conventional rice breeding focus on agronomic traits, such as high yield, high quality (mainly quality), as well as disease and insect resistance. Relatively little research has been conducted on functional nutritional rice in developing countries or research is at the preliminary stages when compared to that in developed countries. Presently, the major challenges associated with functional nutritional rice include the following. Rice breeding research has made three breakthroughs in germplasm resource exploration and utilization. Although there are over 100,000 global rice germplasm resources, those related to functional - nutritional rice are scarce. And the difficulty of cross - country germplasm exchange hinders the rapid breeding of such rice. Second, the molecular basis of research on functional nutritional rice is limited. The identification of genes related to functional nutritional rice is difficult, and the synthesis, metabolic pathways, and molecular regulatory mechanisms of biologically active substances are complex. Furthermore, methods of identifying and analyzing nutritional functional substances are lacking, thereby resulting in high development costs. Third, awareness and acceptance of functional nutritional rice by the public are generally low. The public is not well-informed on functional nutritional rice and they tend to think that it is GM rice. Functional nutritional rice does not have various names, such as functional rice, nutritious rice, selenium-rich rice, which makes it difficult for the public to understand. Although there have been several achievements in rice research, cultivars, and production technologies, the industrialization of nutritional rice is still at an initial stage with small-scale planting and limited market-related products. Influential and widely recognized functional nutritional rice brands are scarce, and key links in the industry chain and value chain are missing. Fourth, the national or industry standards for functional rice need to be improved. To date, no national or industry standards for functional rice and its products have been established, which affects the development of the functional rice industry. Therefore, it is imperative to establish standards, production technology processes, quality evaluation specifications, and clarify relevant labeling for functional nutritional rice.

### 6.2 Prospects

Based on the current progresses of functional nutritional rice, future research should focus on the following aspects. First, strengthening the development and utilization of functional nutritional rice germplasm. A method for identifying functional nutritional rice germplasm from the existing rice germplasm resource library should be established, germplasm with unique biologically active substances should be extensively explored, developed, and utilized. Second, breeding of new functional nutritional rice varieties. Conventional breeding methods should be combined with modern breeding techniques, and molecular design breeding to accelerate the cultivation of new nutritional functional rice varieties. Third, strengthen basic research on nutritional functional rice. Key genes that regulate the metabolic pathways associated with the synthesis of biologically active substances should be explored, and their regulatory mechanisms and metabolic networks analyzed. Effective connection between basic research and breeding applications of nutritional functional rice should be promoted.

In future, the priorities for improving functional nutritional rice should focus on enhancing yield, optimizing plant type, and balancing taste. Understanding regulatory networks and metabolic pathways can lead to more precise and efficient breeding strategies. Due to the low yield and poor taste of commercially available rice varieties, the recognitions of growers and consumers are low. Simultaneously enhancing stress resistance to ensure stable yield and quality under adverse conditions. Strengthen micronutrients, especially iron, zinc and other microelements, to address global micronutrient deficiencies and improve the nutritional status of the population. The cultivation of rice varieties with specific health functions suitable for specific populations should also be a priority research direction.
